# Supplementary material for: Mechanisms of Impact of Alnus ferdinandi-coburgii Odor Substances on Host Location of Tomicus yunnanensis
Source: Insects. 2025 May 23;16(6):553. doi: 10.3390/insects16060553 (PMC12193113; doi:10.3390/insects16060553)
Supplement: Supplementary file 1 [file insects-16-00553-s001.zip › Supplementary Figure S3.docx]

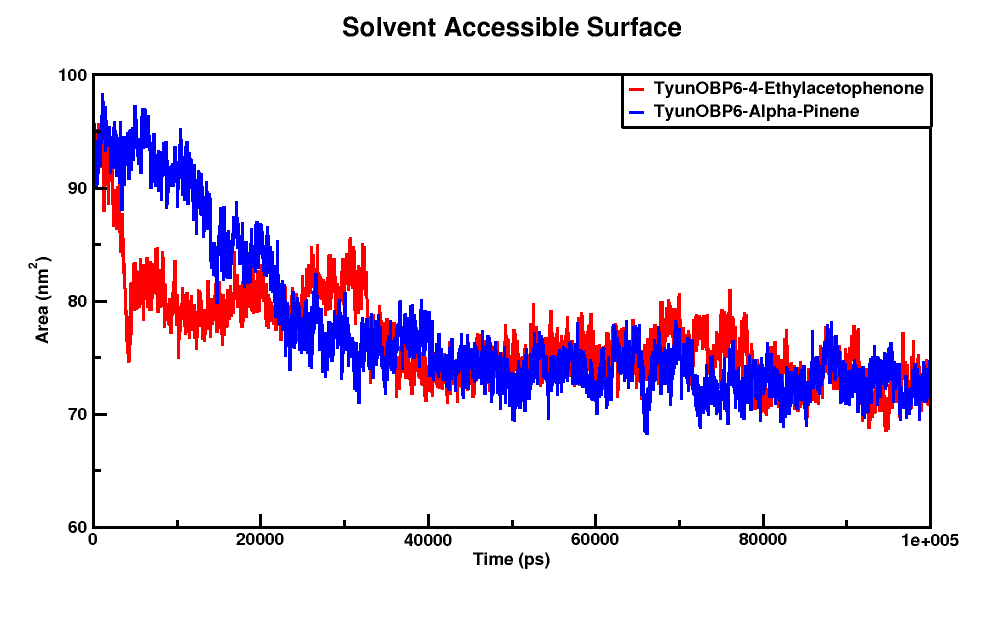


SASA results for TyunOBP6 with ligand


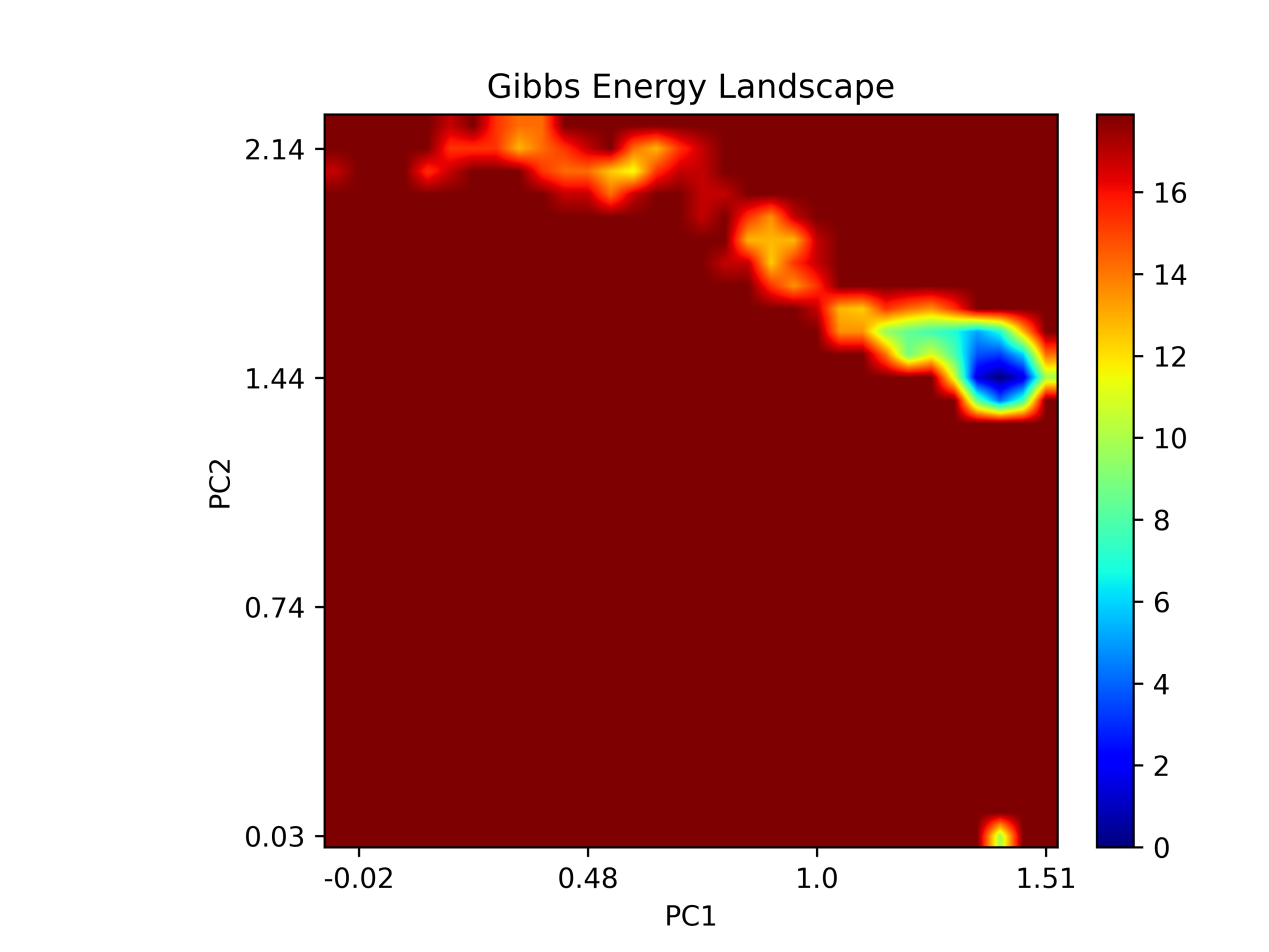


Visualization of TyunOBP6 relative free energy results


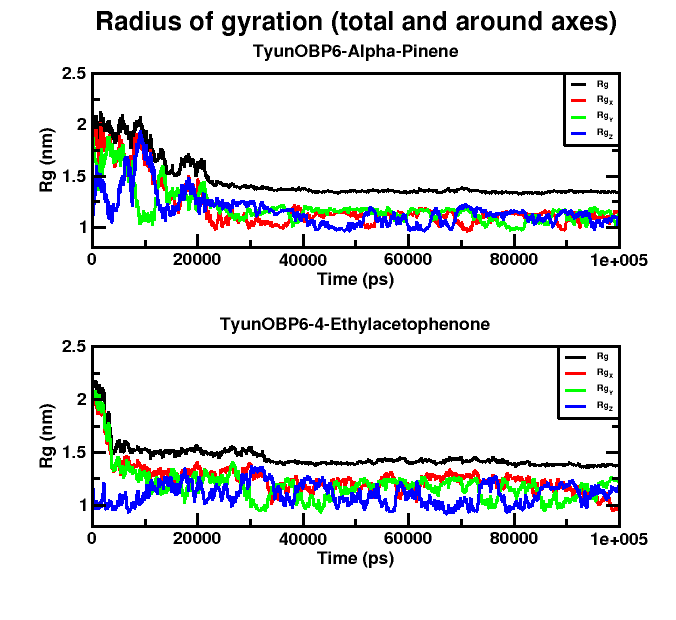


Rg results for TyunOBP6 with ligand


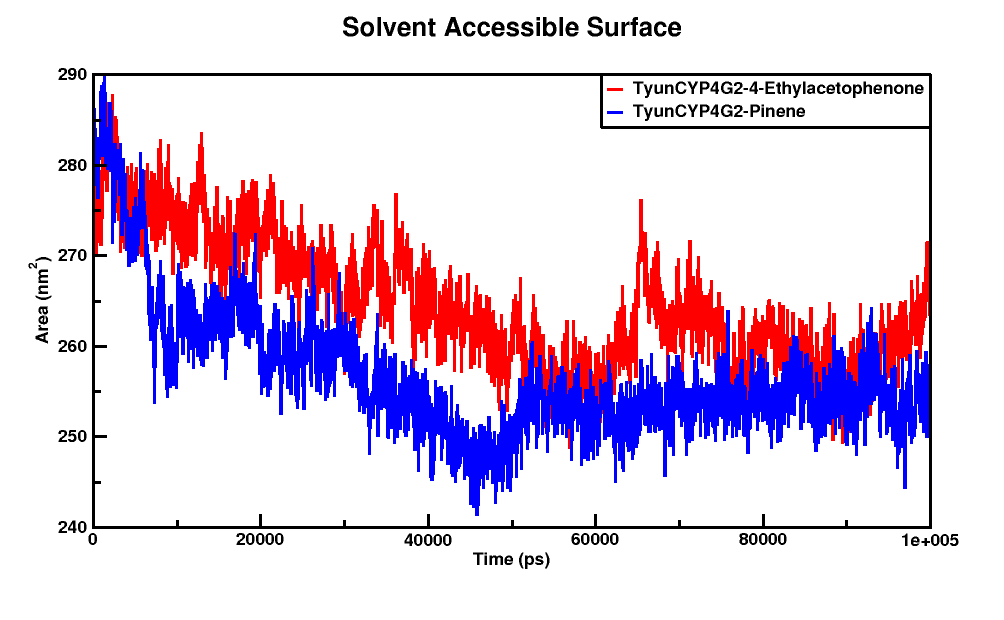


SASA results for TyunCYP4G2 with ligand


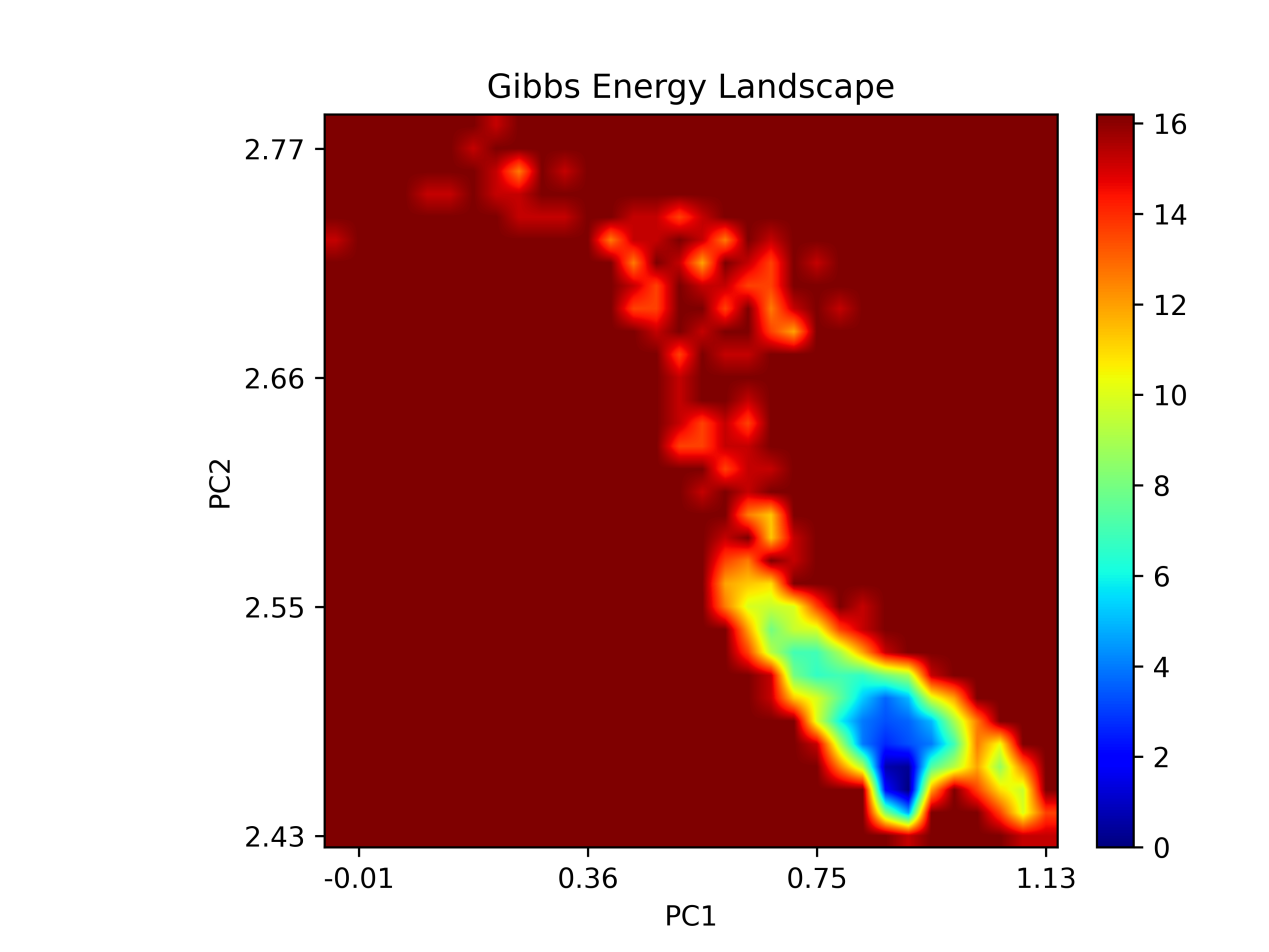


Visualization of TyunCYP4G2 relative free energy results


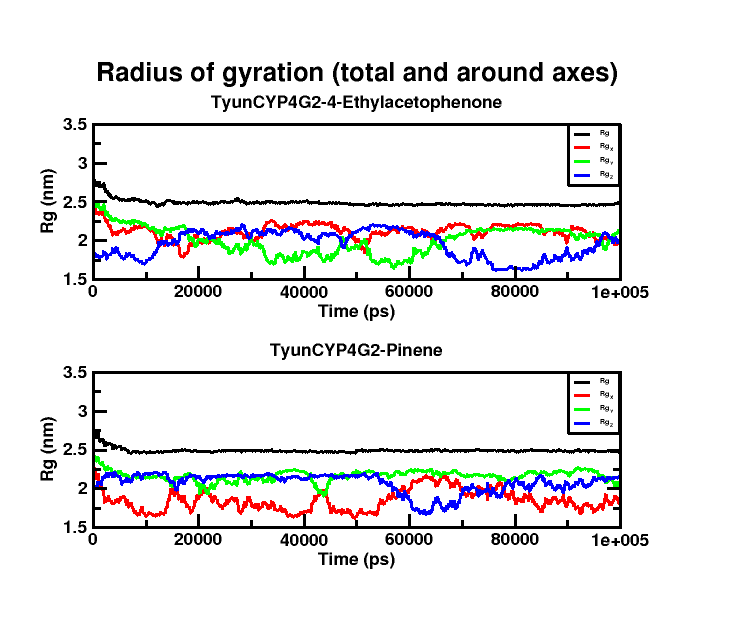


Rg results for TyunCYP4G2 with ligand
