# Supplementary material for: Mechanisms of Impact of Alnus ferdinandi-coburgii Odor Substances on Host Location of Tomicus yunnanensis
Source: Insects. 2025 May 23;16(6):553. doi: 10.3390/insects16060553 (PMC12193113; doi:10.3390/insects16060553)
Supplement: Supplementary file 1 [file insects-16-00553-s001.zip › Supplementary Figure S2.docx]

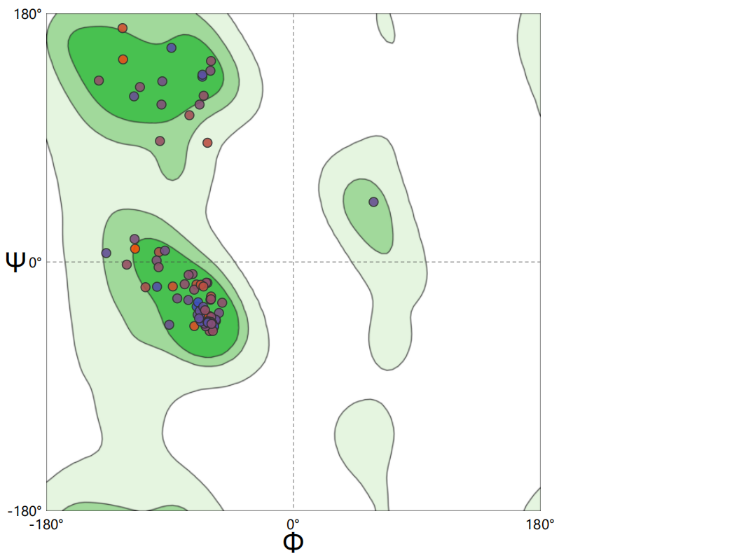


TyunOBP6
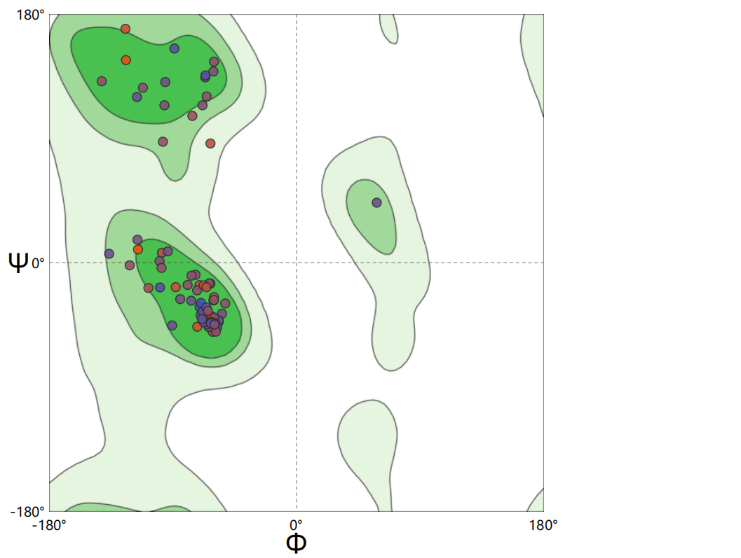

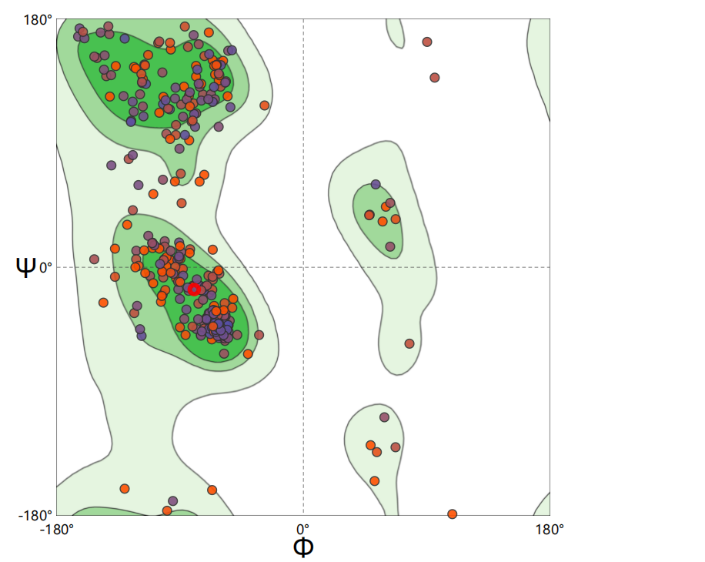


TyunCYP4G2 TyunCYP6DF1

Supplementary Figure S2: The Ramachandran map of the model of three chemosensory related protein from *T. yunnanensis*.
