# Supplementary figures and images for: Mechanisms of Impact of Alnus ferdinandi-coburgii Odor Substances on Host Location of Tomicus yunnanensis
Source: Insects. 2025 May 23;16(6):553. doi: 10.3390/insects16060553 (PMC12193113; doi:10.3390/insects16060553)

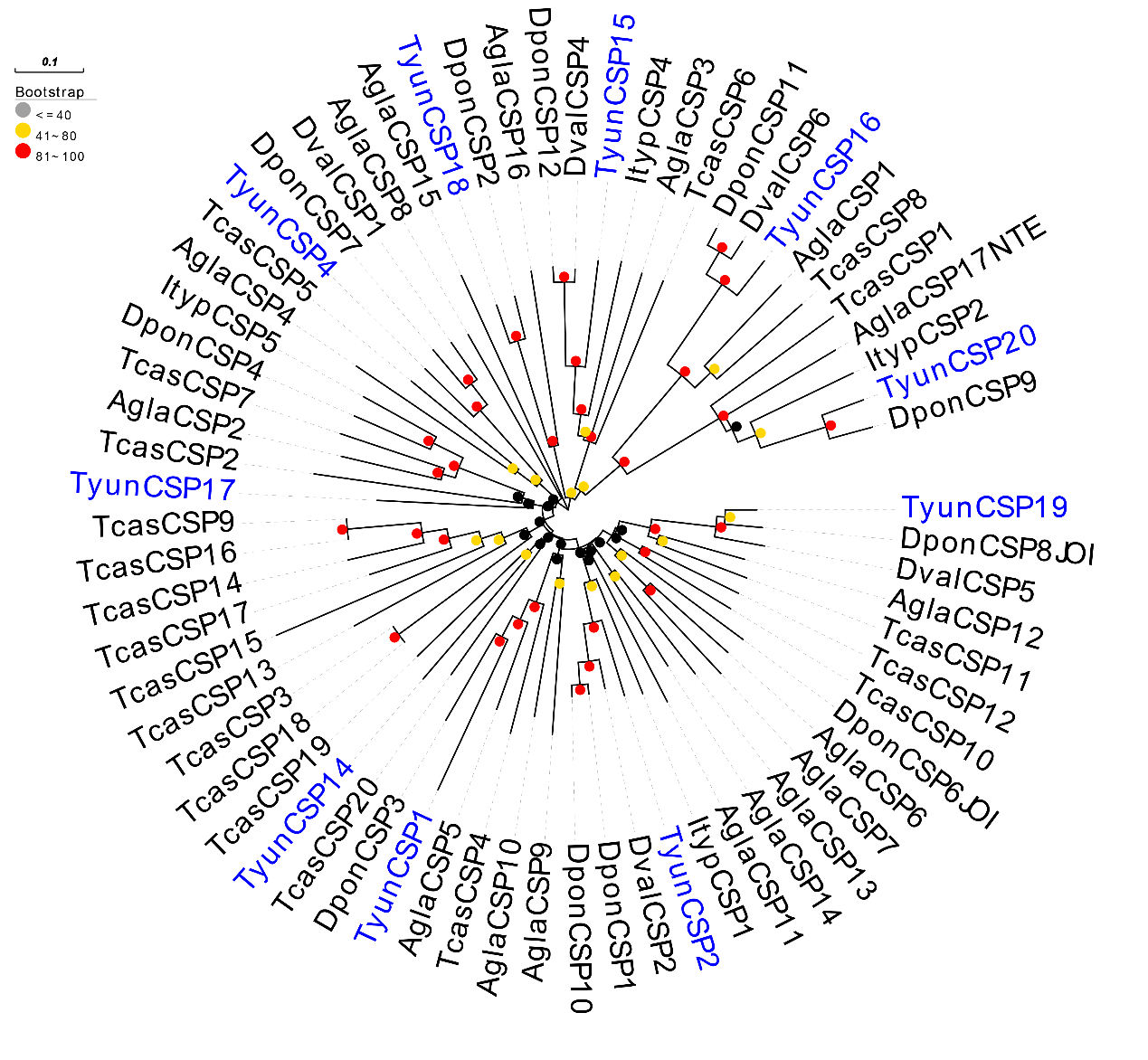

Supplement: Supplementary file 1 [file insects-16-00553-s001.zip › Supplementary Figure S1A.png]

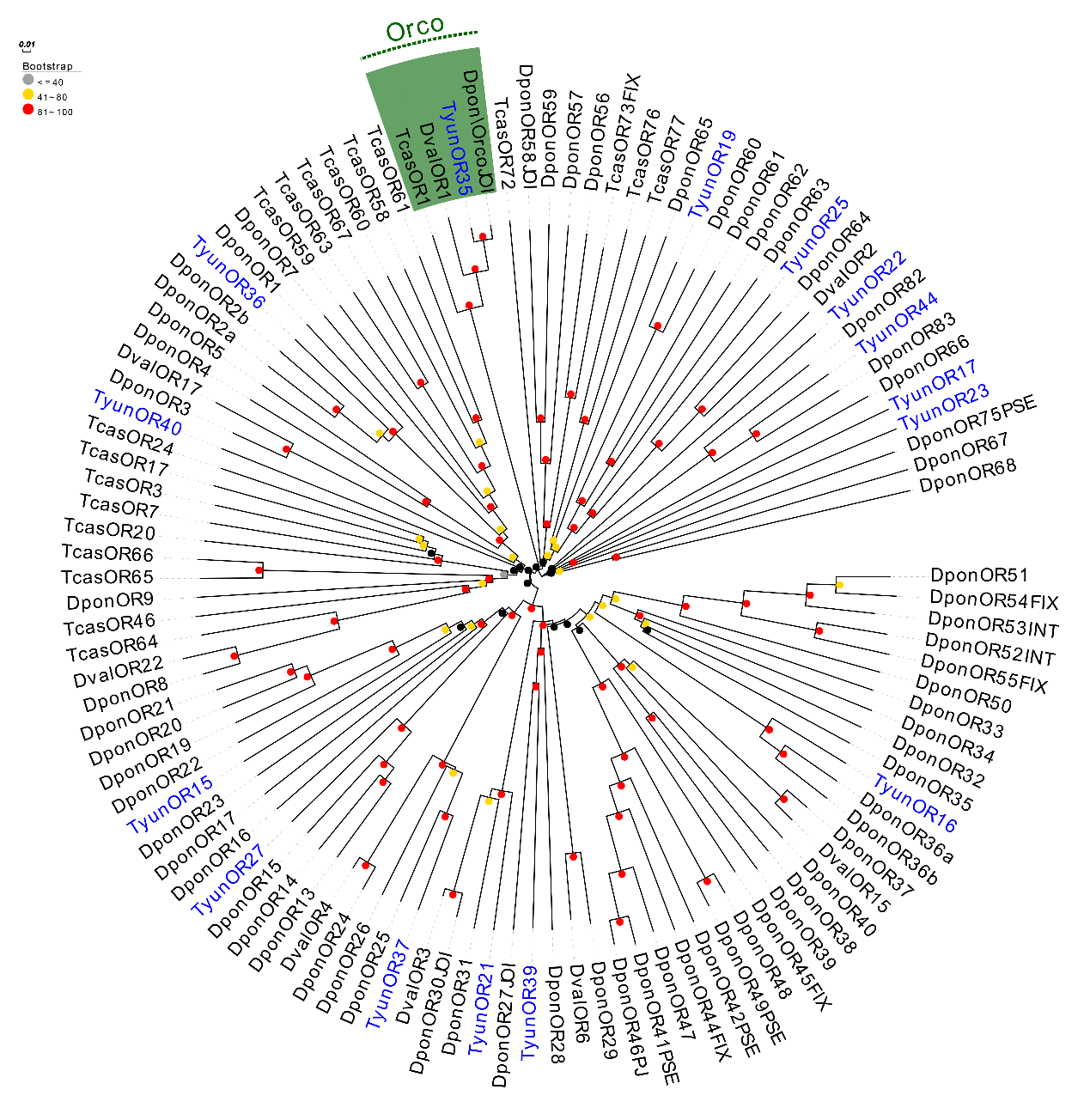

Supplement: Supplementary file 1 [file insects-16-00553-s001.zip › Supplementary Figure S1B.png]

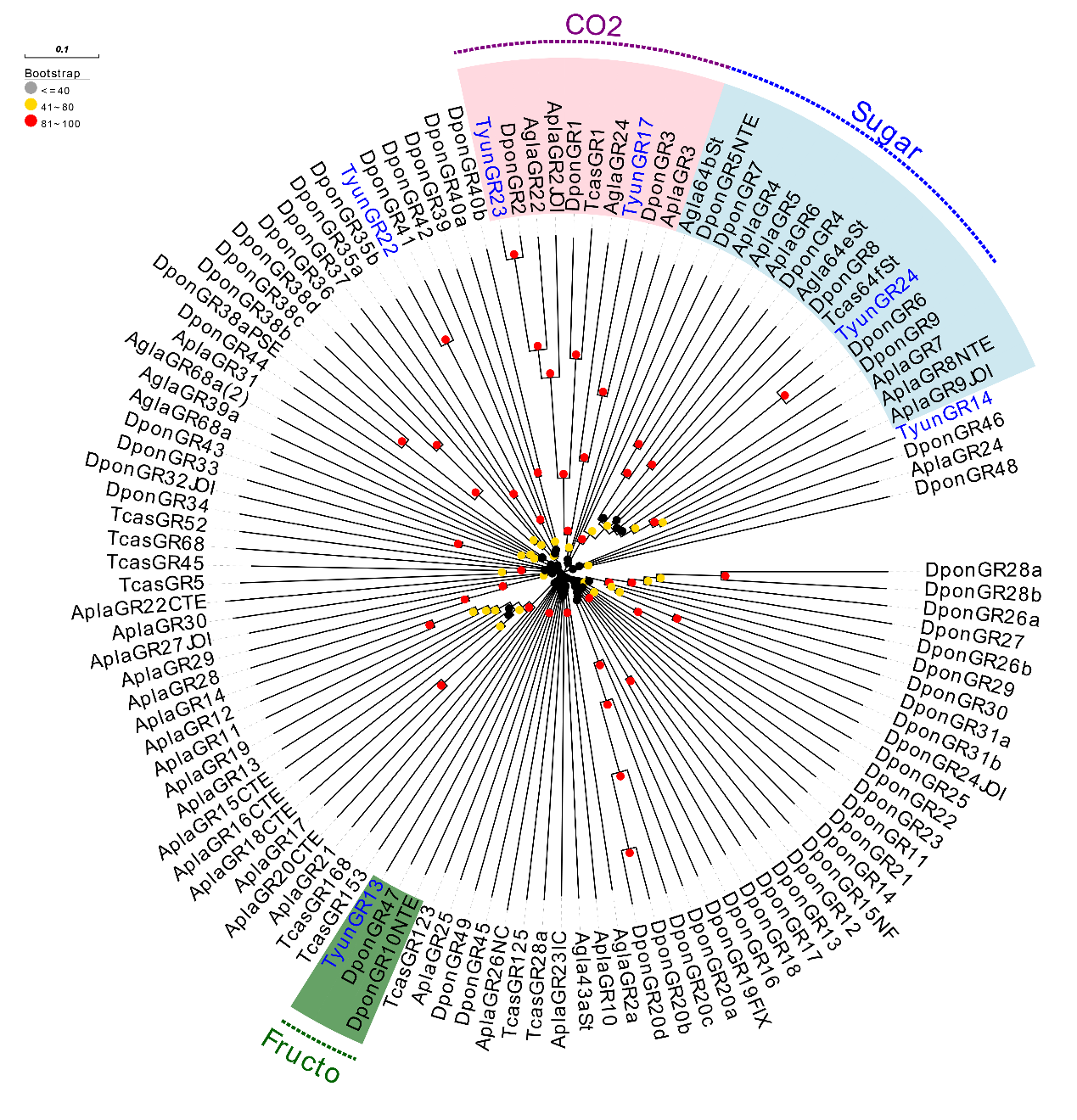

Supplement: Supplementary file 1 [file insects-16-00553-s001.zip › Supplementary Figure S1C.png]

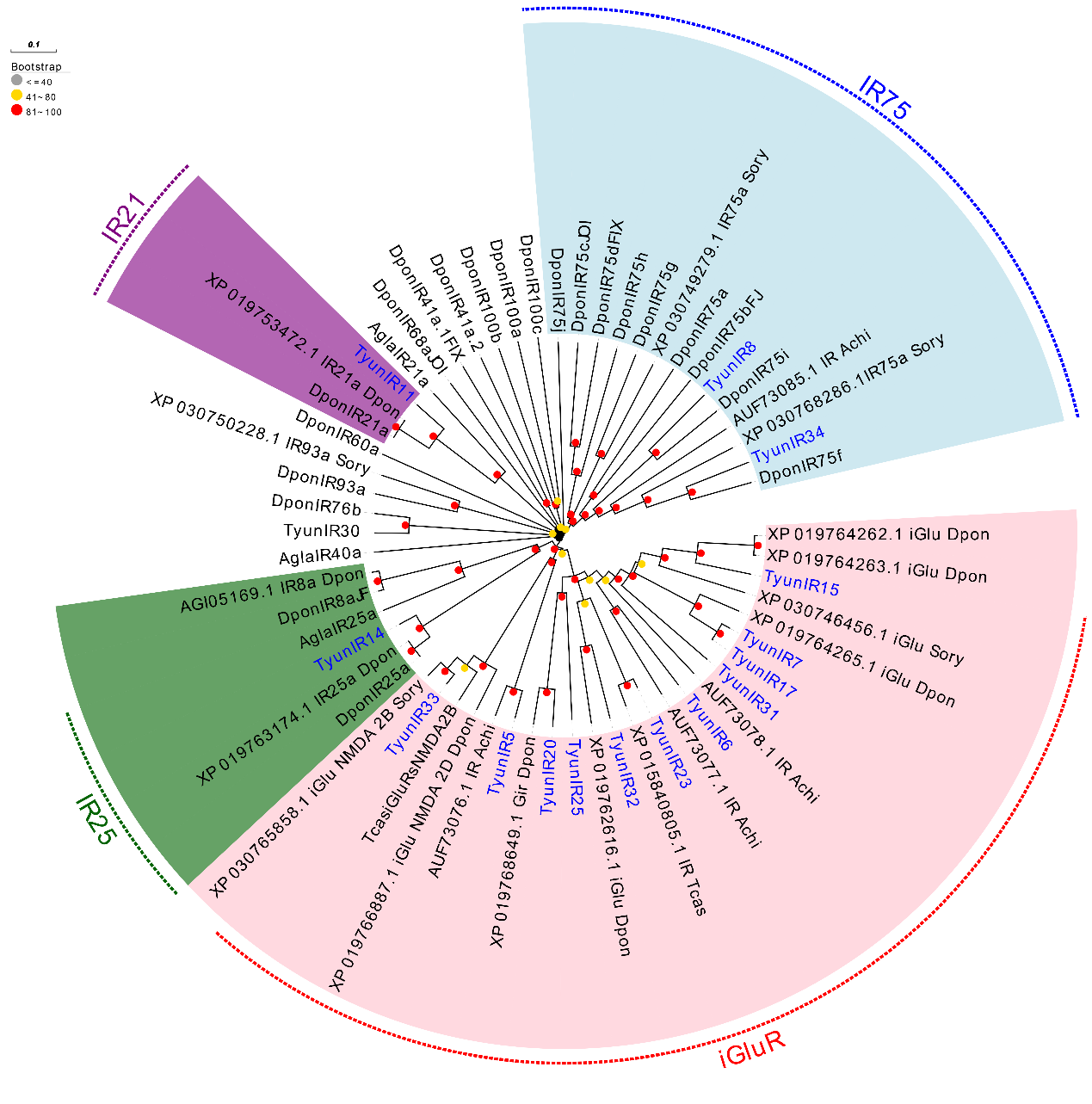

Supplement: Supplementary file 1 [file insects-16-00553-s001.zip › Supplementary Figure S1D.png]

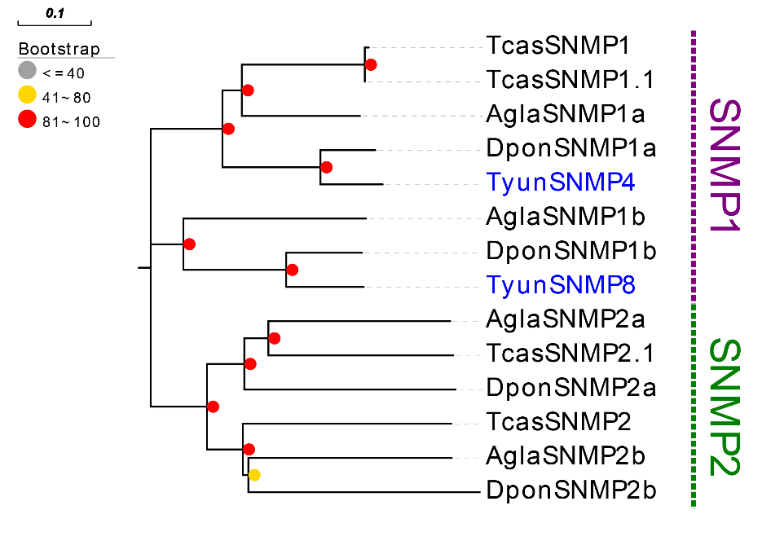

Supplement: Supplementary file 1 [file insects-16-00553-s001.zip › Supplementary Figure S1E.png]
